# Synthesis of TiO_2NWS_@Au_NPS_ Composite Catalyst for Methylene Blue Removal

**DOI:** 10.3390/ma11061022

**Published:** 2018-06-15

**Authors:** Fan Fu, Feifei Wang, Ting Li, Chenlu Jiao, Yan Zhang, Yuyue Chen

**Affiliations:** 1 National Engineering Laboratory for Modern Silk, College of Textile and Clothing Engineering, Soochow University, Suzhou 215123, China; fanfu326@163.com (F.F.); ffwang@stu.suda.edu.cn (F.W.); liting507@yeah.net (T.L.); jiaochenlu2013@163.com (C.J.); 2 Nantong Textile & Silk Industrial Technology Research Institute, Nantong 226314, China

**Keywords:** TiO_2NWS_@Au_NPS_ composite, one step synthesis, catalysis, methylene blue degradation

## Abstract

In this article, HBP-NH_2_-modified titania nanowire (TiO_2NWS_)-decorated Au nanoparticles (TiO_2NWS_@Au_NPS_) were synthesized by one-step method. The role of HBP-NH_2_ concentration in the formation of TiO_2NWS_ was investigated. The fineness and uniformity of pure TiO_2NWS_ were enhanced by absorbed amino groups from amino-terminated hyperbranched polymer (HBP-NH_2_). The morphology and crystal structure of TiO_2NWS_ and TiO_2NWS_@Au_NPS_ were examined by transmission electron microscopy (TEM), X-ray diffraction (XRD), and Fournier transform infrared (FTIR) spectroscopy. The chemical states of gold, titanium and oxygen were analyzed by X-ray photoelectron spectroscopy (XPS). The results showed that at the concentration of HBP-NH_2_ 100 g/L, the mean diameter of TiO_2NWS_ was nearly 72 nm and Au nanoparticles were uniformly distributed on the surface of TiO_2NWS_. The presence of Au_NPS_ improved the photocatalytic properties of TiO_2NWS_ under UV light irradiation. The Au load was believed to improve the utilization rate of the photoelectron and activated the adsorbed oxygen. The obtained TiO_2NWS_@Au_NPS_ decomposed 99.6% methylene blue (MB) after 300 min when subjected to UV light irradiation. After five cycles of the catalyzing process, the TiO_2NWS_@Au_NPS_ still retained over 90% of its catalytic ability for MB. The Au deposition was found responsible for the high catalytic activity of TiO_2NWS_@Au_NPS_.

## 1. Introduction

The constant increase in environmental pollution has raised public concern in recent years. Anthropogenic pollution has so far exceeded the acceptable safety levels to the environment to become dangerous to human health [1,2]. Although great advances have been made in technologies associated with pollutant sources and disposal, many problems still exist when it comes to conversion of polluted waters into harmless products. The textile printing and dyeing industry is one particular field that requires more attention [3,4]. Printing and dyeing wastewaters and heavy metal wastewaters count for more than 70% of all world annual wastewaters. This is far higher in comparison with other pollutions. Therefore, the problem captures lots of interest in both academic and industrial research. For instance, Rehorek et al. studied the degradation of dye using ultrasounds [5]. Wang et al. investigated the catalytic degradation of methyl orange using semiconductor catalysts [6].

Titanium dioxide (TiO_2_) is a promising photocatalyst for environmental application due its high reactivity, chemical stability, low cost, and environmentally friendly features [7]. Titanium dioxide has a conduction band, valence band potential, and gap energy. Despite that, bare TiO_2_ assumes several flaws, such as absorption capacity of ultraviolet wave band due to its higher forbidden band in width (3.2 eV for anatase and 3.0 eV for rutile), as well as less effective photoelectric quantity attributed to photo-generated electron holes [8,9,10,11]. Some studies dealing with the mechanism of filling the gap and strengthening the photocatalytic activities have been reported [1]. Inorganic metallic elements, such as C, S and N, have been used to improve the catalytic activity of TiO_2_. Other materials such as surface precious metal deposition and semiconductors compounding have also been tested [12,13,14]. 

So far, not all methods have been found useful. Precious metal-based catalysts have been the subject of great interest for eliminating pollution due to their high activities and superior stabilities, despite their cost. One attractive feature of metal-based catalysts is the ability of the metals to scavenge photogenerated electrons when subjected to UV-illumination and reduce charge carrier recombination [15]. Gold (Au) has a unique conductivity and magnetic-optical properties, making it an attractive metal for this kind of applications [16,17,18,19]. The method of depositing precious metals normally was prepared by two-step process which required scaffolding work [20,21]. Hence, precious metal modification has constantly been employed in solar cells [22,23].

One-dimensional nanomaterials have received significant advancement in terms of morphology and microstructure. These include nanowires, nanorods and nanotubes, among others. From the photocatalytic perspective, the nanowires possess high specific surface areas which facilitate the electron transport and minimize electron loss at the surface boundary [24,25]. Our group designed poly amide network polymer (HBP-NH_2_) with reticulated porous cavity, which is promising for excellent nanometer reaction container for controlling morphology and size of nanomaterials. This method was applied to the synthesis of silver nanoparticles [26,27]. 

In this work, nanometer-sized titanium dioxide and gold composite catalyst were prepared by the sol-hydrothermal-gel method. The influence of HBP-NH_2_ in nanowires formation was examined. Moreover, the effect of exposure to UV light, TiO_2_ and amount of Au deposited on TiO_2_ surface on catalyst performance were investigated and the results were discussed.

## 2. Experimental

### 2.1. Materials

Tetrabutyl titanate (TBT, Sigma-Aldrich, St. Louis, MI, USA) and HAuCl_4_ (Sigma-Aldrich) were used as catalyst supports in all experiments. All chemicals were of analytical grade and used as received. These included ethylic acid (>99%, Sigma-Aldrich), ethyl alcohol (>99%, Sigma-Aldrich), hydrochloric acid (36%, Sigma-Aldrich), sodium hydroxide (>99%, Sigma-Aldrich), and methylene blue (MB) (>99%, Sigma-Aldrich). amino-terminated hyperbranched polymer (HBP-NH_2_) was synthesized as described in our previous paper [26], and then diluted to 100 g/L for subsequent usage.

### 2.2. Catalyst Preparation

#### 2.2.1. Preparation of Pure TiO_2NWS_

TBT was first dissolved in ethanol at volume ratio of 1:5, and vigorously stirred at ambient temperature until the color changed from colorless to yellow (solution A). Another mixture (solution B) was prepared by mixing glacial acetic acid, deionized water, and ethanol at volume ratio of 1:1:5. Then, solution A was dropped into solution B and evenly stirred at room temperature. The sol was obtained after 12–24 h, which would turn to a hard gel with light blue color.

A certain concentration of HBP-NH_2_ was gently dropped into 10 g of the gel. Afterward, NaOH solution was dropped under constant stirring for 30 min. Finally, the mixture was placed in a 100 mL Teflon-lined autoclave and sealed into a stainless steel tank. The hydrothermal temperature was then elevated to 200 °C for 24 h without shaking or stirring during heating. Next, the autoclave was naturally cooled to room temperature, and the obtained sample was sequentially washed several times with diluted HCl aqueous solution, distilled water, and absolute ethanol to remove any remaining ions. The samples were then dried at 70 °C and the titania nanowires with white color were finally produced. Three kinds of HBP-NH_2_ concentrations (30 g/L, 100 g/L and 200 g/L) were used.

#### 2.2.2. Preparation of TiO_2NWS_@Au_NPS_

All Au-loaded catalysts reported here were prepared by the hydrothermal gel method using TBT as precursor and HAuCl_4_ as Au source. A typical synthesis procedure consisted of dispersing 10 g TBT in 50 mL ethyl alcohol. The obtained mixture was then added drop-wise to the solution which comprised deionized water, ethyl alcohol and ethylic acid at volume ration of 1:5:1. The mixed solution was stirred for 2 h then placed at room temperature until the sols became gels before the hydrothermal reaction in a Teflon-lined stainless-steel vessel. Gels were formed at 200 °C under air oven by mixing tertiary mixture of NaOH, HBP-NH_2_ and HAuCl_4_ (0.05 M), as well as binary mixture of NaOH and HBP-NH_2_.

The hydrothermal reaction conditions were maintained for 24 h, and HBP-NH_2_ was carried out in 100 g/L. The corresponding mole ratios of AuCl_4_^−^ to Ti^4+^ were 5%, 2%, 1% and 0.5%, respectively. After cooling to room temperature, the samples were washed with HCl and deionized water before drying. Also, different samples were used as controls.

### 2.3. Catalyst Characterization

The characterizations of TiO_2_ nanowires were conducted by field emission microscope scanning electron microscopy (FESEM, Hitachi S-4800, Hitachi, Ibaraki, Japan). Transmission electron microscopy (TEM), selected area electron diffraction (SAED), high-resolution electron microscopy (HRTEM) and EDS spectra Ti catalysts were performed on a Tecnai G20 microscope (FEI Company, Hillsboro, OR, USA) operating at 200 kV and equipped with both energy dispersive X-ray spectroscopy system and SIS CCD camera for digital imaging. 

The crystal structures of the samples were determined in air by X-ray diffraction (XRD) using Cu Kα X-ray high source at voltage of 40 kV and current of 30 mA (Philips, Amsterdam, The Netherlands). The data were collected by varying 2Theta between 30° and 90° at step size of 5.0 °/min. Fourier transform infrared spectroscopy (FTIR) measurements were recorded on Nicolet 5700 (Thermo, Madison, MI, USA) at resolution of 4 cm^−1^. X-ray photoelectron spectroscopy (XPS) analyses were performed on a monochromatic X-ray source (AlKα, 1486.68 eV) using VG ESCALAB Mk II. The Ti 2p, Au 4f and O 1s core-level spectra were recorded and their corresponding binding energies were referenced to as C 1s peak at 285 eV (from surface carbon). The core-level spectra were obtained using software package Thermo Advantage after subtraction of Shirley-type background. The total MB contents in the catalysts were analyzed by Cary 50 Bio UV-visible spectrophotometer (Varian, Palo Alto, CA, USA) at wavelength of 665 nm.

### 2.4. Catalysts Activity Testing 

The catalytic activities of the samples were assessed by spiral reactor as described elsewhere [15]. Typical measurements consisted of loading 100 mg catalysts into a beaker containing 50 mL MB. The mixture was then ultra-sonicated for approximately 15 min. The initial concentration of MB was set to 10 mg/L, and catalysts were exposed to UV light (20 W black-light-blue lamp). The suspension was first stirred for 30 min under dark conditions to reach adsorption equilibrium [15]. Next, the suspension was exposed to UV light and stirred for 5 h at the distance of 15 cm between the light source and liquid surface. During pre-treating stage, the solution was fetched 3 mL liquid followed by centrifugation at 9000 r/m for 15 min to separate the catalyst from the solution. The described procedure was followed at regular intervals while UV light was kept on. Afterward, MB concentration was monitored by UV-visible spectrophotometry. With absorption standard curve equations of MB, the concentration of MB (*c*) was obtained by measuring the intensity of characteristic peak located at 665 nm, and the pre-treated concentration was designated as *c*_0_.

To evaluate the recoverability and reusability, the TiO_2NWS_@Au_NPS_ was catalyzed repeatedly for five consecutive cycles under UV light. The catalyzed process was performed in MB solution with the concentration of 10 mg/L with stirring for 4 h. After each cycle, the TiO_2NWS_@Au_NPS_ was alternated with deionized water and washed three times with ethanol, then centrifuged and dried at 80 °C to obtain the recoverableTiO_2NWS_@Au_NPS_

## 3. Results and Discussion 

### 3.1. Catalyst Characteristics

**Figure 1:**
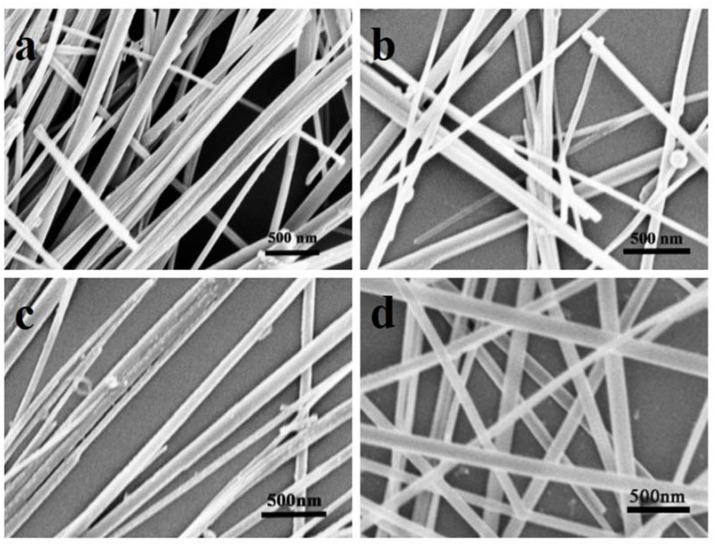
Scanning electron microscope (SEM) images of pure TiO_2_ (**a**) HBP-NH_2_: 0 g/L; (**b**) HBP-NH_2_: 30 g/L; (**c**) HBP-NH_2_: 100 g/L; and (**d**) HBP-NH_2_: 200 g/L.

The respective SEM images of pure TiO_2NWS_ catalysts with four kinds of HBP-NH_2_ concentrates are shown in Figure 1. After 24 h of hydrothermal reaction at 200 °C, the formed nanowires became very copious in quantity. Also, they showed good dispersion without contaminants attached to their surfaces. 

Moreover, from Table 1, HBP-NH_2_ structure in the used concentration range accelerated fineness of TiO_2NWS_ but the effect decreased gradually as HBP-NH_2_ levels rose [26].

For each HBP-NH_2_ concentrate, 100 nanowires were selected randomly to calculate the mean diameter. The results are shown in Table 1. The standard deviation values (σ) were high due to estimating diameters of nanowires by observed sample data. A confidence interval gave an estimated range of values which was likely to include unknown diameters of TiO_2NWS_, the estimated range being calculated from a given set of sample data. In addition, a confidence interval for the mean diameter ($\bar{D}$), based on a simple random sample of size (n = 100), was $\bar{D}\pm z^{*}\frac{\sigma }{\sqrt{n}}$, where $z^{*}$ was the upper (1 − C)/2 critical value for the standard normal distribution. For a confidence interval with level C=95%, the value $z^{*}$ was equal to 1.96. As the sample size increased, the standard error approached the true standard deviation σ for large n. These confidence intervals obtained were presented in Table 1.

**Figure 2:**
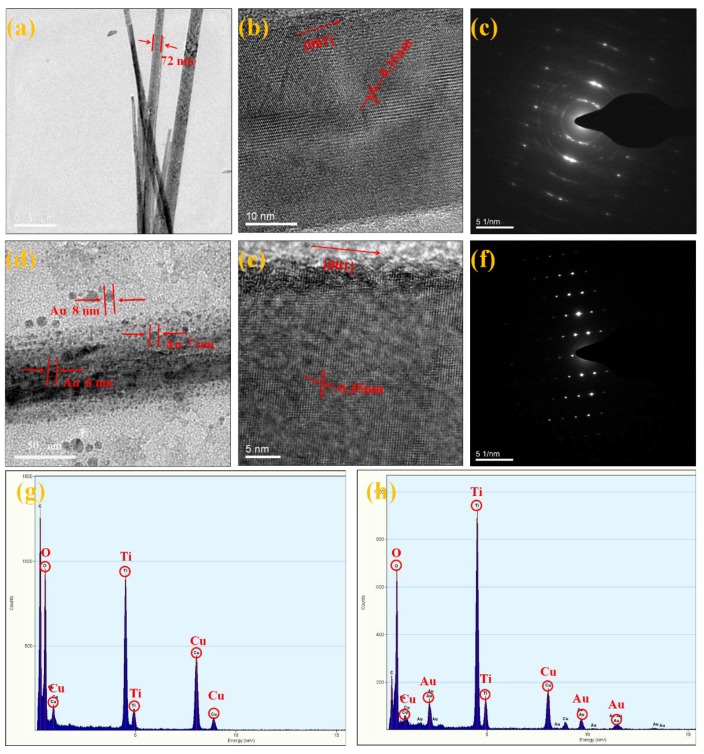
(**a**) Transmission electron microscopy (TEM) image of TiO_2NWS_; (**b**) high-resolution transmission electron microscopy (HRTEM) image of TiO_2NWS_; (**c**) selected area electron diffraction (SAED) image stated of TiO_2NWS_; (**d**) TEM image of TiO_2NWS_@Au_NPS_; (**e**) high-resolution transmission electron microscopy (HRTEM) image of TiO_2NWS_@Au_NPS_; (**f**) selected area electron diffraction (SAED) image stated of TiO_2NWS_@Au_NPS_; (**g**) EDS of TiO_2NWS_; and (**h**) EDS of TiO_2NWS_@Au_NPS._

It could be seen that the diameter of the nanowires decreased first with the increase of HBP-NH_2_ concentration. At the concentration of 100 g/L, the mean diameter was nearly 72 nm. However, the mean diameter increased when the HBP-NH_2_ concentration increased to 200 g/L. Therefore, the mean diameter of pure TiO_2NWS_ can be controlled by the concentration of HBP-NH_2_. Thus, 100 g/L of HBP-NH_2_ was selected to prepare TiO_2NWS_@Au_NPS_. TEM was used to characterize the surface morphologies of TiO_2NWS_ and TiO_2NWS_@Au_NPS_. Figure 2a estimated the average diameters of TiO_2NWS_ to around 72 nm, and with high purity surface. Figure 2d depicted that Au deposits with very small sizes (<8 nm) were formed and distributed all over the TiO_2NWS_ support surface.

Figure 2b,e represent high-resolution transmission electron microscopy (HRTEM) images of Figure 2a,d, respectively. The plane intervals were measured as multiples of 0.35 nm, representing stripe images of (101) plane of anatase TiO_2_. The structural features of these materials were also observed by Chemseddine and Moritz [28] and were linked to cluster-cluster growth and condensation of skewed octahedral for anatase. Also, the crystals were grown along the (001) direction, marked with an arrow. Figure 2c,f displayed selected high crystallinity images of TiO_2NWS_ and TiO_2NWS_@Au_NPS_ obtained by selected area electron diffraction (SAED) which indicates that the crystal has an anatase phase crystalline structure [29]. Figure 2g represents the EDS of TiO_2NWS_, which was mainly composed of Ti and O. Figure 2h depicts the EDS of TiO_2NWS_@Au_NPS_, which was mainly composed of Ti, O, and Au (Cu was copper during measurement). 

**Figure 3:**
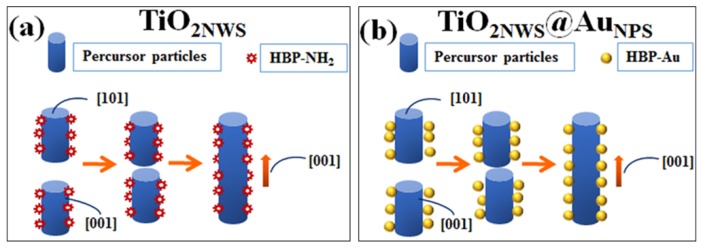
(**a**) Schematic diagram for the synthesis route of TiO_2NWS_ and (**b**) schematic diagram for the synthesis route of TiO_2NWS_@Au_NPS_ composite.

The formation mechanism of the nanowires can be explained by Figure 3a. Some studies reported that the surface density of six-fold-coordinated Ti-atoms with hydroxyls on (001) surface structure was much higher than the (101) surface [30,31]. The number of hydroxyl groups was estimated to 7.0 nm^−2^ for (001) surface and 5.1 nm^−2^ for (101) surface [32]. The (001) plane adsorbed much more HBP-NH_2_ than the (101) plane for hydrogen bonds formed by hydroxyl and amino groups. This also hindered the contact between (001) planes. As shown in the schematic model, oriented attachment on the (101) plane could occur, attributed to high concentrations of HBP- NH_2_ which would restrict the growth of cross-sections.

The formation of TiO_2NWS_@Au_NPS_ is schematically shown in Figure 3b. During the initial stage of hydrothermal reaction, AuCl_4_^−^ was deoxidized into Au [27]. The amino-rich surface of Au particles made them easy to be attached to precursor particles by hydrogen bonding. This induced Au-load precursor grown through the (101) surface, together with formation of nanowires and TiO_2NWS_@Au_NPS_ in one step in situ method_._

**Figure 4:**
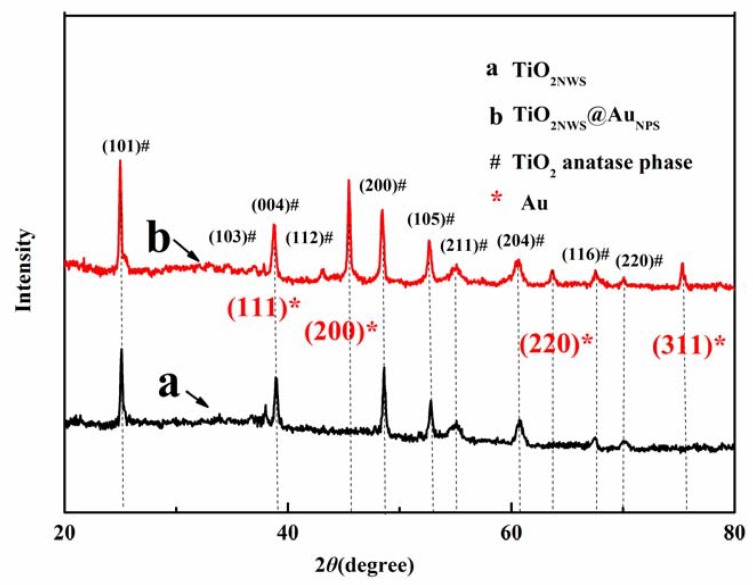
X-ray diffraction (XRD) patterns of pure TiO_2NWS_ and TiO_2NWS_@Au_NPS_.

Figure 4 depicts the XRD patterns of pure TiO_2NWS_ and TiO_2NWS_@Au_NPS_ nanocomposite prepared by one step in situ hydrothermal method. The XRD pattern contained line (a) corresponding to pure TiO_2NW_ and line (b) of TiO_2NWS_@Au_NPS_. Both samples showed 6 distinct peaks, indexed as (101), (004), (200), (105), (211) and (204) lattice planes (black hash symbol). By consulting the standard color card (JCPDSPDF#: 021-1272), no rutile or irrelevant diffraction peaks were observed [33,34]. The crystal shapes of the diffraction peaks looked clear and sharp, indicating well-formed crystals of the material in anatase. In addition, the (111), (200), (220) and (311) lattice planes (red five-point star) belonged to Au, which were uniformly distributed on TiO_2NWS_ surface. This suggested the successful formation of gold particles in the composites, which were dispersed on TiO_2NWS_ surface [35]. No other lattice planes were found in (103), (004) and (112), which may be due to the proximity to strong lattices of Au (111) that overlapped each other. The (200) crystal surface of Au was brought into the Scherrer Formula: ($D=\frac{K\gamma }{B\cos\theta }$), and the calculated mean diameter of Au_NP_ was estimated to 7 nm. The diameter agreed with TEM results.

**Figure 5:**
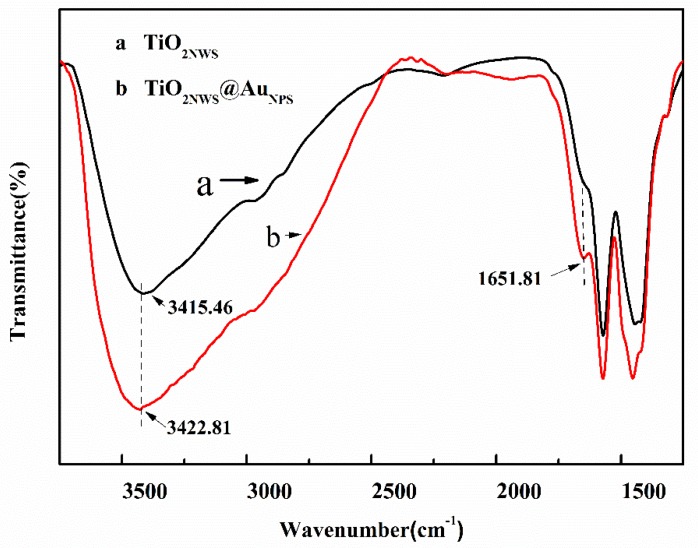
FTIR spectra of pure TiO_2NWS_ and TiO_2NWS_@Au_NPS_.

The FTIR spectra of TiO_2NWS_ and TiO_2NWS_@Au_NPS_ catalysts collected under ambient conditions were gathered in Figure 5. Different sharp transmittance peaks of TiO_2NWS_ and TiO_2NWS_@Au_NPS_ appeared at approximately 1650 cm^−1^ and 3410 cm^−1^. At wavelengths from 1200 cm^−1^ to 3650 cm^−1^, the spectra demonstrated changes in infrared transmission characteristics resulting from deposition of Au on titanium dioxide supports. The strong absorption peak of line (a) and line (b) at 3200–3250 cm^−1^ was assigned to gamma O-H stretching vibration of hydroxyl groups (Ti-OH) present on the materials. This could be water molecules accompanied by the surface of titanium. In addition, the shift in transmission of TiO_2NWS_@Au_NPS_ was due to generation of additional energy levels within the band gap of TiO_2NWS_ and overlapped original TiO_2_ band gap excitation with plasmon band of Au nanoparticles [30]. A narrow transmittance peak was apparent for O-H bending vibration of TiO_2NWS_ particle surface hydroxyls at 1652 cm^−1^, which slightly increased as gold load increase. Compared to pure TiO_2NWS_, the band signal increased the transmission coefficient, suggesting the composite generated more active O-H groups. Based on FTIR analysis, the density of hydroxyl of TiO_2NWS_ was lower than that of TiO_2NWS_@Au_NPS_, which was important to the photocatalytic activity.

**Figure 6:**
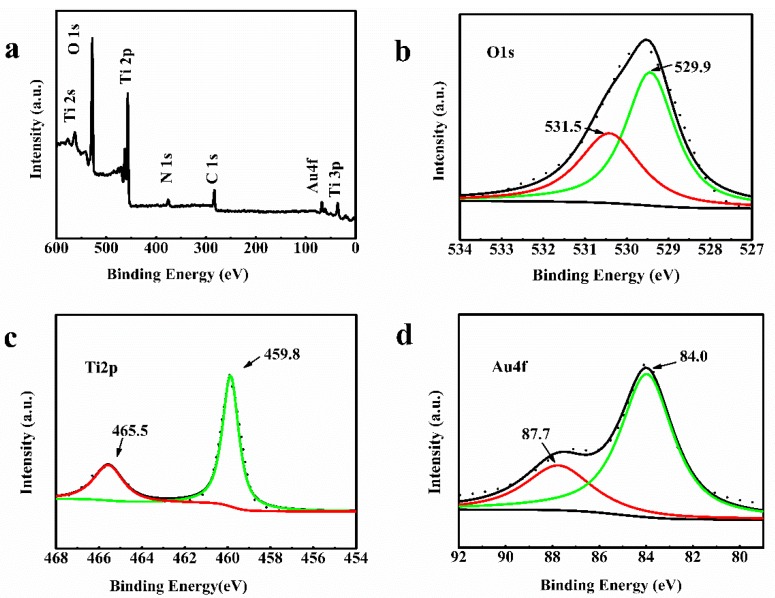
XPS spectrum of TiO_2NWS_@Au_NPS_ (**a**); Core-level spectra of O 1s (**b**); Ti 2p (**c**); and Au 4f (**d**).

To further explain the differences in reactivities between both Au-loaded and pure TiO_2NWS_, XPS analyses were conducted to investigate the surface oxidation states of Ti 2p, Au 4f, and O 1s core levels. The XPS results are summarized in Figure 6a and the original spectra are provided in Figure 6b–d. The XPS profiles of TiO_2NWS_@Au_NPS_ showed binding energy peaks of C, Ti, O, N, and Au (Figure 6a). The presence of Au peak indicated the successful immobilization of Au_NPS_ on TiO_2NWS_. The C 1s and N 1s were attributed to the incomplete removal HBP-NH_2_. The high–resolution Ti 2p XPS spectrum in Figure 6c showed two binding energy (BE) peaks at 465.5 eV and 459.8 eV, corresponding to Ti 2p_1/2_ and Ti 2p_3/2_ spin-orbit in TiO_2NWS_, respectively. After Au doping, the Ti position shifted to high energy of about 1.2 eV, resulting from electron in titanium dioxide oxygen void transferred to the gold particles [36]. Furthermore, the distance between the two peaks was nearly 5.7 eV, indicating that titanium existed in the crystal lattice as Ti^4+^ [37,38,39]. The O 1s XPS spectrum showed TiO_2NWS_@Au_NPS_ a lower binding energy of 529.78 eV (Figure 6b), characteristic of Ti-O bond. Also, a higher binding energy of 531.25 eV characteristic of O–H bonds appeared [40,41,42]. Figure 6d showed the high electron spectrum of Au 4f region. The two BE peaks at 84.0 eV and 87.7 eV corresponded to Au 4f_7/2_ and Au 4f_5/2_ spin-orbit energies [43]. The distance between the two peaks was 3.7 eV, meaning that the samples contained one state of Au, namely Au0. In addition, all HAuCl_4_ was reduced [44]. The pattern showed clear peaks at positions and locations of binding energies matching reported values.

### 3.2. Catalytic Performance

To investigate the catalytic activity of the TiO_2NWS_@Au_NPS_ during photocatalytic reactions, the degradation of MB was conducted under UV. The photodegradation efficiency calculated as $\eta {=(c/c}_{0})\text{×}100\%$, where *c* is the concentration of MB after certain catalysis time and c_0_ represents the pre-treated concentration. 

**Figure 7:**
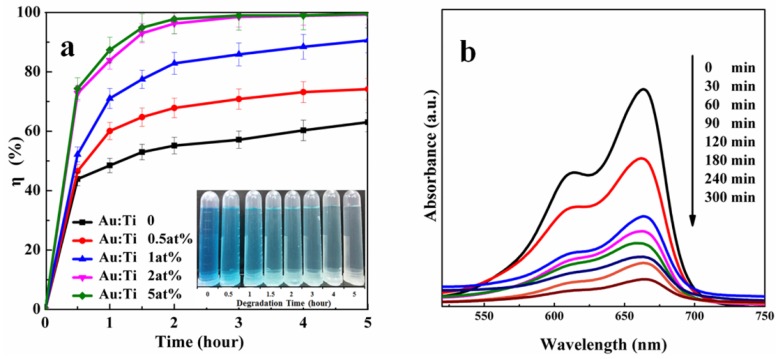
(**a**) MB photodegradation efficiency of materials under UV light irradiation and changes in color during MB degrade process; (**b**) UV-absorption spectra during MB degrade process.

The photodegradation efficiencies after 5 h of UV light irradiation are presented in Figure 7a. The photodegradation efficiency decreased in the following order: TiO_2NWS_@Au_NPS_ (5 at% Au) > TiO_2NWS_@Au_NPS_ (2 at% Au) > TiO_2NWS_@Au_NPS_ (1 at% Au) > TiO_2NWS_@Au_NPS_ (0.5 at% Au) > neat TiO_2_. The MB photodegradation efficiencies using pure TiO_2NWS_ and different Au-based photocatalysts were estimated to be 63.06%, 74.19%, 90.69%, 99.3%, and 99.6%, respectively. Obviously, gold complexes displayed higher photodegradation efficiencies than pure TiO_2NWS_. Furthermore, the degradation effect of samples increased as mole rate of Ti rose. This implied that Au played an important role in MB degradation. Furthermore, the degradation efficiency was higher than particle doping with a gold test result [45]. The corresponding color change using Au:Ti 1 at% is also shown in Figure 7a. The color started to fade after 5 h, indicating the degradation of MB. 

Additionally, TiO_2NWS_@Au_NPS_ (1 at% Au) suspension UV-absorption spectra were recorded during the degradation process (Figure 7b). The absorption peak intensity of MB decreased gradually as irradiation time rose accompanied by hypochromic shift from 665 nm to 610 nm. The peak intensity sharply reduced with time after 60 min and became stable after 300 min. The hypsochromic shift suggested that the degradation of MB underwent a series of demethylation processes [46]. Based on the experimental results, chromophoric groups of MB were conjugated in the nitrogen–sulfur system, representing the N-methyl group in the benzene ring. The group corresponded to a wavelength at 665 nm [47]. The presented concentrations of MB in solution were estimated by measuring the maximum absorption peak intensity at 665 nm. During degradation, the conjugated nitrogen–sulfur system became destroyed, and wavelength peak intensity decreased.

### 3.3. Photocatalytic Mechanism

**Figure 8:**
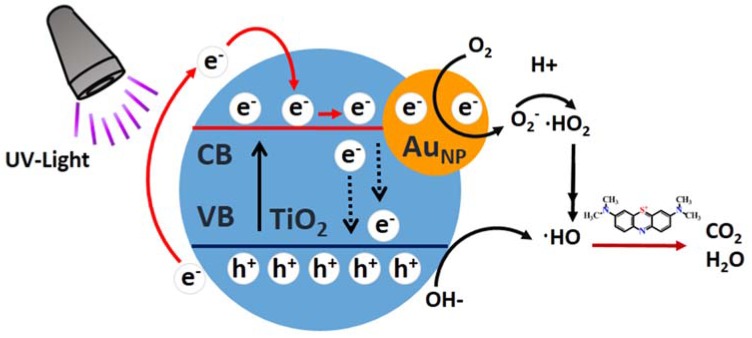
Schematic representation of the mechanism of catalytic MB under UV light.

Based on the above findings and associated discussions presented previously, a schematic depiction of possible reaction mechanisms of the catalytic process was presented in Figure 8 (with dark pre-treatment). Under UV light, electrons in the valence band (VB) of TiO_2_ moved to the conduction band (CB), leaving holes at the valence band (VB). These electrons then reduced O_2_ in TiO_2_ to O_2_^−^ anion radicals, with hole oxidation OH to ·HO free radical. Next, they moved to CB of TiO_2_ to be further trapped by neighboring Au_NPS_. Afterward, the electrons reduced O_2_ present on Au_NPS_ to O_2_^−^. These radicals could receive more electrons to become ·HO. Meanwhile, holes in VB oxidized water present on TiO_2_ to induce ·HO free radicals. Next, ·HO would oxidize MB to form degradation products. Consequently, the synergic effect of oxidation states of gold, including two BE peaks at 87.7 and 84.0 eV (Figure 7d), significantly enhanced the photocatalytic performance of TiO_2NWS_@Au_NPS_. Moreover, FTIR results showed the formation of more OH species following Au loading. This was believed to occur during the leading step to increase ·HO free radicals on the TiO_2_ surface. On the other hand, the crystal structures of Au nanoparticles and TiO_2NWS_ were different, and hence defects and crystal barriers were inevitable at the interfaces of TiO_2NWS_ and Au nanoparticles. Therefore, tremendous defects and barriers existed in Au_NP_. The presence of few interfaces of Au particles could significantly reduce recombination of electrons and holes [48]. Conversely, pure TiO_2NWS_ did not provide distinct transfer paths for electrons, causing easy recombination of electrons and holes. Furthermore, the electrons in the CB can transfer from TiO_2NWS_ to Au_NPS_, resulting from a Schottky barrier being formed at the metal-semiconductor interface [49]. It may be lead to the photo-induced electrons being trapped by Au_NPS_ under UV irradiation, and electrons could reduce O_2_ in TiO_2_ to O_2_^−^ anion radicals. Therefore, low recombination of photogenerated carriers and high photocatalytic activity made TiO_2NWS_@Au_NPS_ provide valuable scaffold for many potential applications, such as photo electrocatalysts, solar cells, hydrogen generation by water splitting, and sensors.

### 3.4. Reusability of TiO_2NWS_@Au_NPS_

**Figure 9:**
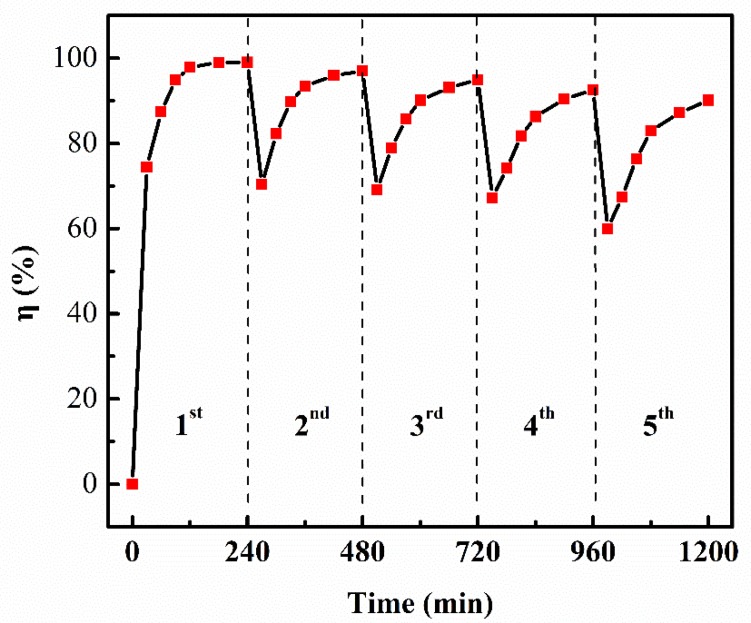
Cyclic catalysis of TiO_2NWS_@Au_NPS_ degradation of MB dye.

Reusability is an essential parameter for potential practical application and further analysis of the mechanism of the catalysis between the catalyst and pollutant. The reusability of TiO_2NWS_@Au_NPS_ (5 at% Au) was investigated by operating the catalyzation process for five consecutive cycles and the degradation amount at each cycle was detected. As displayed in Figure 9, the degradation efficiency for the MB was still above 90% of their initial amounts in the 5th regeneration cycle. Thus, the TiO_2NWS_@Au_NP_ catalyst can be recycled almost without obvious loss in the catalytic performance, demonstrating a benign renewable catalyst.

## 4. Conclusions

A new method to synthesize TiO_2NWS_ and TiO_2NWS_@Au_NPS_ was provided. Insights into the generating role of key nanowires under different conditions were established. Under hydrothermal conditions, the concentration of HBP-NH_2_ played an important role in size and uniformity of obtained TiO_2NWS_ and TiO_2NWS_@Au_NPS_. The results also indicated that within the load of Au_NPS_, the reaction rate improved during degradation of MB under UV light. 5 at% Au of TiO_2NWS_@Au_NPS_ showed the highest level of degradation efficiency, and the lowest degradation efficiency rate was recorded with pure TiO_2NWS_. The Au deposits on TiO_2NWS_ played a major contribution in UV light catalytic activity. The surface-active electron was identified as the primary active species for improving the catalytic reaction. These facts are crucial in defining the catalytic activity in the presence of ultraviolet light. After five cycles of the catalyzation process, the TiO_2NWS_@Au_NPS_ can retain over 90% of its catalytic ability for MB, demonstrating an efficient and benign renewable candidate for pollutant removal

## Figures and Tables

**Table 1:** The relationship between the concentration of the HBP-NH_2_ and the average diameters of TiO_2NWS_.

Concentration of HBP-NH2 (g/L)	Average Diameter ($\bar{D}$) (nm)	Standard Deviation (σ) (nm)	Confidence Interval (nm)
0	97.35	45.21	±8.86
30	89.45	41.48	±8.13
100	71.93	35.21	±6.90
200	79.19	39.30	±7.70
